# Being an Informed Consumer of Health Information and Assessment of Electronic Health Literacy in a National Sample of Internet Users: Validity and Reliability of the e-HLS Instrument

**DOI:** 10.2196/jmir.5496

**Published:** 2016-07-11

**Authors:** Gül Seçkin, Dale Yeatts, Susan Hughes, Cassie Hudson, Valarie Bell

**Affiliations:** ^1^ University of North Texas Denton, TX United States; ^2^ Texas Woman's University Denton, TX United States

**Keywords:** health literacy, health information technology, Internet, information, ehealth

## Abstract

**Background:**

The Internet, with its capacity to provide information that transcends time and space barriers, continues to transform how people find and apply information to their own lives. With the current explosion in electronic sources of health information, including thousands of websites and hundreds of mobile phone health apps, electronic health literacy is gaining an increasing prominence in health and medical research. An important dimension of electronic health literacy is the ability to appraise the quality of information that will facilitate everyday health care decisions. Health information seekers explore their care options by gathering information from health websites, blogs, Web-based forums, social networking websites, and advertisements, despite the fact that information quality on the Internet varies greatly. Nonetheless, research has lagged behind in establishing multidimensional instruments, in part due to the evolving construct of health literacy itself.

**Objective:**

The purpose of this study was to examine psychometric properties of a new electronic health literacy (ehealth literacy) measure in a national sample of Internet users with specific attention to older users. Our paper is motivated by the fact that ehealth literacy is an underinvestigated area of inquiry.

**Methods:**

Our sample was drawn from a panel of more than 55,000 participants maintained by Knowledge Networks, the largest national probability-based research panel for Web-based surveys. We examined the factor structure of a 19-item electronic Health Literacy Scale (e-HLS) through exploratory factor analysis (EFA) and confirmatory factor analysis, internal consistency reliability, and construct validity on sample of adults (n=710) and a subsample of older adults (n=194). The AMOS graphics program 21.0 was used to construct a measurement model, linking latent factors obtained from EFA with 19 indicators to determine whether this factor structure achieved a good fit with our entire sample and the subsample (age ≥ 60 years). Linear regression analyses were performed in separate models to examine: (1) the construct validity of the e-HLS and (2) its association with respondents’ demographic characteristics and health variables.

**Results:**

The EFA produced a 3-factor solution: communication (2 items), trust (4 items), and action (13 items). The 3-factor structure of the e-HLS was found to be invariant for the subsample. Fit indices obtained were as follows: full sample: χ^2^ (710)=698.547, *df*=131, *P*<.001, comparative fit index (CFI)=0.94, normed fit index (NFI)=0.92, root mean squared error of approximation (RMSEA)=0.08; and for the older subsample (age ≥ 60 years): χ^2^ (194)=275.744, *df*=131, *P*<.001, CFI=0.95, NFI=0.90, RMSEA=0.08.

**Conclusions:**

The analyses supported the e-HLS validity and internal reliability for the full sample and subsample. The overwhelming majority of our respondents reported a great deal of confidence in their ability to appraise the quality of information obtained from the Internet, yet less than half reported performing quality checks contained on the e-HLS.

## Introduction

Technological advancements inevitably change the information dissemination process by creating new information outlets and developing a platform for new sources [1]. The emergence of Web 2.0 has changed the way consumers interact with technology, information, and health providers. Electronic health, health-related Internet-based technology, and information and communication technologies are broad terms encompassing an array of electronic and mobile phone apps that uses the Internet to deliver health and medical information, independent of space and time considerations often associated with more conventional sources of information. People use desktops, laptops, tablets, and smart phones to access information. These technologies are closely interwoven with the medical field altering self-health care behavior by transforming the scope, breadth, and pace with which information is obtained [2,3]. A study performed in the United States in 2012 found that 81% of the Internet users searched the Web for health information [4], with the majority looking for information about a specific condition or disease [5]. According to the 2014 Pew Internet survey, approximately 1 in 4 people with a chronic illness have read someone’s posting about a health issue on a website. Over 70% of people in Europe access health information on the Internet [6]. Studies have also reported that the Internet-based information has a strong effect on how people manage their health. Specifically, Americans often turn to health information on the Internet before seeing a health professional [7]. In fact, people now use the Internet more often than consulting with their doctors [8]. Underlying the growing use of the Internet to gather information is a willingness to become involved in health care decision making and the ability to make informed choices and decisions [9]. As Dutta-Bergman stated, “the critical role of the Internet as a health information resource has shifted traditional patterns of consumer health information use, the physician-patient relationship, and health services delivery” [10]. Numerous scholars have discussed the transformative effect of the Internet on our self-care transforming patients into a reflexive consumer who can make informed decisions. Ehealth information resources have empowered patients to make informed decisions by improving their ability to communicate with their health care providers [11-13].

The term ‘‘e-patients’’ was coined to describe individuals who are empowered by various technology-based health information tools and apps, but concerns persist about information accuracy, credibility, and quality [14]. Considerable health information available on the Internet is of varying quality; much of it may be oversimplified, incomplete, inaccurate, or misleading [15]. Although it has been shown that patient–physician interactions can prove more satisfactory thanks in part to better informed patients, nearly 60% of ehealth seekers report that they have hesitated talking to their providers about information from the Internet due to fears of straining their relationship with their physician [16]. Moreover, most people fail to apply any criteria to assess the quality of Web-based information, and instead, they trust that source is credible [17]. Complicating the issue is the fact that according to the Institute of Medicine, nearly 90 million Americans have low health literacy, adversely affecting their ability to appraise health information before making and implementing health care decisions [18]. Most Internet health information searches are generally conducted through a general search engine, accessing a multitude of websites of varying quality. Not surprisingly, as Web-based sources of information proliferate, people report increasing confusion and uncertainty about the quality of information available [19]. Fortunately, there are a number of general guidelines for appraising the credibility and quality of ehealth information, including measures of content accuracy, the provision of disclosure statements, and the currency of information, which constitute ehealth literacy skills. Unfortunately, most users are more influenced by the design and appeal of a website when determining its trustworthiness [12]. Consequently, this raises the concern that ehealth seekers might engage in behavioral practices that might be harmful or dangerous to their health. Information overload, sifting through vast amounts of information while simultaneously trying to decipher its quality has been described as mindboggling and may lead to negative affect, such as fear or anxiety.

Although Internet use may lead to a sense of patient empowerment, empowerment without the requisite high level of health literacy may pose a health risk should a patient misuse the information or decide there is no real need to see a doctor [20]. Thus, the American Medical Association and the National Committee on Quality Assurance have recommended ehealth literacy as one of the top areas for national action. The emphasis on the importance of health promotion and patient self-care in maintaining health and well-being, and a partnership with providers via access to information technology, has led to increased professional discourse on the value of ehealth literacy [18-22]. As the role of digital information technologies in health research continues to unfold, it is necessary to examine the synergy between the multidimensional factors associated with health literacy and their effects on self-health care outcomes [23].

The Institute of Medicine considers health literacy to represent a “constellation of skills” necessary to act on health care information [18]. The lack of an integrated theoretical framework has led researchers to operationalize health literacy in different ways, leading to limited progress in understanding and measuring health literacy [24,25]. Traditionally, health literacy was defined as an individual’s capacity to obtain, process, and understand basic health information and the services needed to make appropriate health decisions [18]. The US National Assessment of Adult Literacy defined health literacy as ‘‘the ability of U.S. adults to read, understand, and apply health-related information presented in written English to function in society and achieve one’s goals.’’[26]. Ratzan et al, define health literacy as “[t]he degree to which individuals can obtain, process, understand, and communicate about health-related information needed to make informed health decisions” [3]. Sørensen’s definition encompasses the individual’s competence to appraise health information to make judgments concerning personal health care [26]. These definitions provided a foundation for a concept of health literacy that evaluated reading and medication-related numeracy skills. Several other researchers’ definition includes communication with health care providers [27-28]. Thus, health literacy is associated with self-care, health promotion and wellness, and better navigation of the health care system, all of which are important for regaining and maintaining health [29-30]. Health literacy is particularly salient to the aging population’s increasing longevity. Older adults develop substantial health care needs as they age, and research examining this demographic group’s health literacy is much needed. Moreover, the burgeoning focus on successful aging strategies and quality of life for the ageing has provided a greater impetus for understanding their capacity to make informed appraisals of Internet health information [31,32]. Notwithstanding, despite the continual evolution of health literacy, there has been a research lag in establishing multidimensional instruments to measure the construct [33]. In our research, we emphasize the importance of measuring ehealth literacy because most existing measures fail to target the health literacy skills of ehealth information seekers despite its potential impact on a range of health outcomes [9,33]. As Pleasant stated “many people have offered many definitions, yet those definitions have not been formally or fully tested. While health literacy evaluative and measurement tools often claim to be based on one definition or another, the specific constructs within the definitions have rarely, if ever, been explicitly built into and tested with the evaluative tool.’’ [34]. Furthermore, the definition of health literacy needs to be broadened [35]. In the age of electronic health information, measures that reflect an ability to read static text still predominate. Norman emphasized the need for research to develop a valid self-report measure that assesses ehealth literacy skills due to the expansion of health information available on the Internet [36]. There are few tools to assess users’ ability to engage in ehealth in an informed way [37-38]. ‘‘Lacking empirical evidence of the relationship between different literacy skills, reading and numeracy skills are often used as proxies of literacy in research and practice’’ [39]. However, the ehealth websites require a consumer to have the ability to appraise the content of informational resources to make health decisions. “Accessing health information had never been easier than in the current information age as the Internet’s vast content and global reach allows health consumers to quickly connect with the latest information’’ [40]. Although health literacy for written resources is well defined and various measures exist, the evaluation of health literacy in a digital context is less clear. Obtaining health information from the Internet can be very helpful to those who are able to discern the difference between reliable and unreliable health information websites. Accordingly, a new generation of health literacy assessment tools, as a response to the new digital technologies, is needed. The measurement of health literacy should be adjusted to reflect technological changes [41].

Our goals in this research were twofold: (1) to develop a tool to be used in ehealth literacy research and to examine its psychometric properties; and (2) to help understand how ehealth literacy is associated with health care variables. Our ehealth literacy measure is a tool designed to assess the degree to which people possess the skills required to use ehealth information in an informed way. Originally, Norman and Skinner introduced the concept of ehealth literacy, defining it as the ability to seek, find, understand, and appraise health information from digital sources and apply this knowledge to solve health problems. Their Lily Model included 6 core health literacy skills depicted as petals of a lily: traditional (reading ability and numeracy), information, media, health, computer, and science [41]. According to Jordan et al, most of previous instruments assessed user competency with Web technologies; however, they failed to capture user skills required in the age of ehealth information through the Internet. They have also been found to have substantial psychometric weaknesses [42]. These measures, such as the Rapid Estimate of Adult Literacy in Medicine and the Test of Functional Health Literacy in Adults among others, assess operational skills (basic skills needed to use the Internet), formal skills navigation, information skills (locating information), logic skills (ability to understand information), functional literacy (reading and understanding health information), and strategic skills (applying information to health problems) [43-49]. The 8-item eHealth Literacy Scale designed by Norman and Skinner measures a consumer’s perceived skills at using information technology such as their comfort in using computers and ability to locate health information [41]. Although these instruments address a combination of technical aspects related to the use of the Internet and content provided, they do not measure ability to appraise health information. A research study reported that almost 90% of the participants in a discussion of health literacy agreed that current measures of health literacy do not match with the current understanding of health literacy in age of information technology [24]. Understanding ehealth literacy requires an examination of critical issues such as the users’ ability to find appropriate information and use it to gain better control over their personal health. Although current assessments of health literacy focus primarily on reading ability, our review of the literature suggested the need for updated measures of health literacy that would measure information search strategies and skills to judge the quality of information found [12,50,51]. Current research instruments fail to capture important aspects of ehealth literacy such as appraisal, trust, and the communicative aspects of it as an interactive process. To address this gap, we designed items to reflect these components of ehealth literacy.

This study advances this effort by developing an ehealth literacy scale for users of digitally provided health information. Most existing measures of health literacy focus on a single dimension, which tends to be a reading comprehension test emphasizing a relatively narrower cognitive capacity to understand health-related texts and materials [24]. The need to navigate health websites with confidence is particularly important because the consequences for using low-quality, misleading, or false information could endanger health and possibly result in death [35]. Through our review, we identified key attributes of ehealth literacy demands. An area of consensus is evaluating information to discern high-quality information from low-quality information. Accordingly, our measure and its items reflect this area of consensus.

## Methods

### Data Source and Sample

The sample consists of respondents who used the Internet for health information (N=710). The Knowledge Networks (KN), a nonprofit academic research firm, recruited the respondents who are members of the first Web-based panel representative of the US population. The KN Panel consists of about 50,000 US residents, aged 18 years or older. The KN uses an address-based sample frame derived from the US Postal Service Delivery Sequence File, which covers 97% of US households, thereby maximizing sample representativeness. Address-based sampling permits probability-based sampling of addresses including those households that have unlisted telephone numbers, do not have landline telephones, do not have Internet access, and do not have devices to access the Internet. Respondents are randomly selected, in contrast to the opt-in convenience sampling design of most other Web-based panels. The KN Panel members who were randomly selected were invited to become panel members. For those selected households that do not have Internet access or devices to access the Internet, we provided a Web-enabled computer with free Internet service to enable their participation as Web-based panel members. The KN obtained the participants’ consent before they become panel members [52].

For this study, 1315 participants were randomly selected after being contacted via an email. Potential participants were prescreened through the question “Do you seek health or medical information on the Internet for yourself and for others?” We obtained a 70% response rate and received a total of 710 completed Web-based questionnaires. The Web-based survey consisted of 50 questions. It was self-administered and accessible for a designated period of time. Respondents were able to complete the survey only once. The 19-item electronic health literacy measure was developed through an extensive multistep scale development and evaluation process. We created items based on a review of the literature. Approval of the Institutional Review Board of the University of Maryland, Baltimore County, was also obtained (protocol number: Y11GS21145) before the study’s launch. During the pilot phase of our project, we tested general readability and item wording. We field-tested the items (n=10) to assess clarity of wording and general readability of the items and whether participants interpreted the items as we intended. No problems were reported by our pilot study respondents in regard to clarity of survey questions.

An inherent part of any survey is nonresponse. The KN attains a 65% to 70% survey completion rate as opposed to 2% to 16% for opt-in Web-based panels. Our specific survey sample was drawn at random from the panel members who were randomly recruited in accordance with scientifically accepted sampling theory and methods. Accordingly, our specific survey sample represents a simple random sample from the larger probability-based panel designed to be statistically representative of the US population. Because all KN Panel households were selected randomly with a known probability of selection and because our survey-specific panelists were then also randomly selected from the larger panel, our results can be interpreted with the statistical confidence relative to the population of the United States [52].

Furthermore, the KN states,

in certain cases, a survey sample calls for pre-screening, that is, members are drawn from a subsample of the panel. There are also several sources of survey error that are an inherent part of any survey process, such as non-coverage and non-response due to panel recruitment methods and to inevitable panel attrition. We address these sources of sampling and non-sampling error by using a panel demographic post-stratification weight as an additional adjustment based on demographic distributions from the most recent data from the Current Population Survey (CPS). This weighting adjustment is applied prior to the selection of any client sample from KnowledgePanel, and these weights are used in the stratified, weighted, selection procedure for drawing samples from the panel. All the above weighting is done before the study sample is drawn. Once a study sample is finalized, a set of study-specific post-stratification weights are constructed so that the study data can be adjusted for the study’s sample design and for survey nonresponse. Starting with each panel member’s base weight, an iterative raking procedure is used to achieve an optimal approximation of the relevant benchmarks to make survey respondents representative [52].

### Statistical Analysis

Our psychometric analyses started with exploratory factor analysis (EFA) using principal component analysis and varimax rotation to identify these theorized latent dimensions represented in the variables and to define the underlying structure among the variables. This enabled us to have an initial confidence in our conceptualization. As Hair et al [53] wrote “[e]xploratory factor analysis can be performed to provide a preliminary check on the number of factors and the pattern of loadings. Then proceed to a confirmatory test of measurement theory (to establish the construct validity of the newly designated scale).’’ We examined how many factors existed, whether factors were correlated, and which variables best measured each factor. This also enabled us to determine whether any underlying structure existed for measures on the 19 variables. Hair et al [53] wrote, CFA cannot be conducted appropriately unless the researcher can specify both the number of constructs that exist within the data to be analyzed and which specific measures should be assigned to each of these constructs. After performing EFA, we proceeded with confirmatory factor analysis (CFA) to determine whether the items in our instrument support the 3-factor structure, which provided evidence that the item measures taken from our sample represent the true score that exists in the population. Beginning analytical procedures with EFA by examining the measurement model followed by CFA was also reported in the literature on the psychometric validation of new instruments [54-57]. The factor structure proposed by EFA on the full sample was validated with a subsample comparison approach using a sample of older adults (age ≥ 60 years). This enabled us to assess the stability of the factor structure of the electronic Health Literacy Scale (e-HLS).

We, then, proceeded with CFA for the full sample and subsample. First, the AMOS graphics program was used to construct an input path diagram representing the measurement model that linked the ehealth literacy factors (latent variables) with e-HLS indicators. The model included covariances between the 3 factors, previously proposed by EFA. Data were entered for 710 cases, standardized beta coefficients were generated for all regressions of indicator variables on factors that were included in the model, and the covariance between the factors were obtained. The R^2^ values for all 19 e-HLS indicators were also generated. We repeated these analytical procedures for our subsample by entering data for 194 cases separately from the full sample. The chi-square significance test and overall model fit indices were estimated including the comparative fit index (CFI), the normed fit index (NFI), and root mean squared error of approximation (RMSEA).

Item total correlations and Cronbach alpha internal consistency reliability coefficients were calculated for the full sample and subsample. We tested the validity of our scale by examining its correlations with respondents’ demographic characteristics: age, gender, race or ethnicity, marital status, education level, and income. We performed ordinary least squares regression analyses to examine our scale’s construct validity. We regressed the composite scale on variables in our dataset that are conceptually and empirically related to health literacy. These variables were as follows: perceived empowerment, health interactions, health communication, experiencing health problems, noncompliance, and negative effect. All linear regression analyses were controlled for demographic covariates. Mean replacement procedure was used when missing data are less than 2% of responses for an item. We used SPSS 21.0 in our analyses.

### Measures

Our measure, its dimensions, and the items representing the dimensions were constructed from a literature review of health literacy materials in the Medline, PsycInfo, ERIC, Sociological Abstracts, and Web of Science databases. We also reviewed existing instruments developed for print and Web-based health information materials. We conducted a comprehensive literature review to identify key skills associated with health literacy. In this literature review, we examined how literacy demands of digital health information materials are related to evaluation of information quality. Our review revealed that most existing tools target traditional health literacy for print resources. Given this constraint, we decided to create items based on our review of the literature. We generated items to operationalize each of the 3 conceptual domains identified in the literature: trust, action and behavior. Because the concept of ehealth literacy is increasingly conceptualized as consisting of skills related to evaluating, communicating, and using that information to make informed decisions, we designed our item to reflect these skills.

To measure participants’ trust in the Internet-based sources of health information, actions they take to evaluate information, and the extent to which they engage in informational exchange with health professionals, we asked them to indicate their agreement with the items of our measure. The theoretical basis for the trust items is literature on trustworthiness of Web-based health information such as the California Health Care Foundation’s report and other related literature [58-62]. Scale items designed to measure the communication dimension are based on the findings of previous studies of patient–provider dialogue [63-65]. The items we theorized to represent the action dimension are derived from a review of literature on uses of the Internet for health information and how Internet users evaluate information including the Medical Library Association’s guidelines and other related publications [66-70]. The following is a list of specific items we used in our research:

*Demographic and socioeconomic covariates* included age, race or ethnicity, gender, marital status, education level, and income. Age was measured as an ordinal variable. Gender was coded as (0) male and (1) female. Response categories for race or ethnicity and marital status were collapsed to account for small cell sizes and were measured as dichotomous variables. Race or ethnicity was measured as (0) Caucasian and (1) minority. Education level was coded as (1) high school or less, (2) some college or associate degree, (3) college degree, and (4) postgraduate degree. Annual family income was categorized into 4 groups: (1) $29,999 or less, (2) $30,000 to $59,999, (3) $60,000 to $99,999, and (4) $100,000 and above. Marital status was measured as (0) married and (1) nonmarried.

*Electronic health literacy* was measured with our scale, which we labeled e-HLS. It is a 19-item self-report scale that examines the (1) behavioral, (2) communicational, and (3) attitudinal components of health literacy among ehealth information seekers. Each item was rated on a 5-point Likert scale ranging from 1=“never or strongly disagree” to 5=“always or strongly agree.” The survey assessed whether ehealth information seekers do the following when gathering information from the Internet: (1) read disclosure statements on health websites; (2) check for credentials and institutional affiliations of those who provide information on websites; (3) check the ownership of a health website; (4) check a website’s sponsor(s); (5) check for financial ties between website information and the website’s sponsor(s); (6) appraise the adequacy and integrity of information providers’ credentials; (7) check to see whether a physical address is provided; (8) check for stated goals and objectives; (9) appraise whether coverage of health topics is clear and comprehensive; (10) check whether other print or Web resources confirm information provided; (11) checked whether information is current and updated; (12) check for the last time information was updated. We also asked (13) if they were confident in their ability to appraise information quality on the Internet; and if they (14) asked health professionals for advice about where to find credible information on the Internet; (15) discussed information obtained from the Internet with a health professional; (16) believed information provided on the Internet was credible; (17) believed information provided on the Internet was balanced and accurate; (18) thought information provided on the Internet was the same as or better than what most health professionals provided; and (19) trusted the Internet for obtaining accurate health information. We reverse-coded the last 4 items so that lower scores represent greater consistency with awareness of varying quality of health information. Our scale had a Cronbach alpha coefficient of .93.

All our questionnaire items had equal weight and were measured on the same metric, a 5-point Likert measurement scale. This ensured that none of the items were more influential than the other items in averaging an overall score for our scale. Consistent with the literature, we calculated a score for each subscale that used items with different response options and performed separate reliability and validity analyses for each. DeCoster stated, “[y]ou might create a group of items to determine respondents' opinions on each of these issues. Sometimes a single questionnaire contains items from several different scales mixed together. This is perfectly legitimate. In this case your items making up different subscales will be slightly different’’ [71]. In fact, it is not unusual for instruments with subscales to include items with different response options. The expectation is that the direction of the magnitude of the responses between items should be consistent throughout the scale. In other words, questions should be written to indicate that higher scores should indicate more positive responses or greater magnitude on the variable and vice versa [56,71].

*Positive health interaction* was measured by asking respondents to indicate the extent to which they agreed with the following statements: (1) “I receive more attention to my questions from health providers as a result of gathering information from the Internet,” (2) “I receive more information from health providers as a result of gathering information from the Internet,” and (3) “Interactions with health providers have become more respectful as a result of gathering information from the Internet.” Response options ranged from 1=“strongly disagree” to 5=“strongly agree.” The Cronbach alpha reliability coefficient is .87.

*Strained Health Interaction* was measured by asking respondents to indicate the extent to which they agreed with the following statement: “Interactions with health providers have become strained as a result of bringing in health and medical information from the Internet to my appointments.” Response options ranged from 1=“strongly disagree” to 5=“strongly agree.” We first reverse-coded the item and included it with the rest of positive health interaction items, but, we found the Cronbach alpha reliability value to be less than the threshold value of .70. Alpha if item deleted analysis suggested dropping this strain item. Thus, we separated it from the rest of health interaction items and performed a single-item analysis.

*Health communication* was measured through questions that asked respondents to indicate the extent to which they agree with the following statements: (1) “Information on the Internet helps me to communicate more effectively with health providers during appointments,” (2) “Information on the Internet helps me to ask more informed questions to health providers,” and (3) “Information on the Internet helps me to better understand what my health provider is telling me during appointments.” Response options ranged from 1=“strongly disagree” to 5=“strongly agree.” The Cronbach alpha reliability is .88.

*Nonadherence* was assessed through the following questions: (1) “Do you change your willingness to accept a health care provider’s treatment after reading information on the Internet?,” (2) “Do you doubt diagnosis or treatment of a health care provider if it conflicts with information on the Internet?,’’ and (3) “Have you ever changed a health care provider’s treatment as a result of information obtained from the Internet?” Response options ranged from 1=“never” to 5=“always.” The Cronbach alpha reliability coefficient for this measure is .71.

*Perceived empowerment* was assessed with a single item that asked respondents to indicate the extent to which they agreed with the following statement: “Gathering information from the Internet about my health makes me feel empowered.” The response options ranged from 1=“strongly disagree” to 5=“strongly agree.”

*Negative effect* was measured with a statement that asked respondents to indicate the extent to which they agreed with the following statement: “Gathering information from the Internet about my health makes me worried and/or anxious.” The response options ranged from 1=“strongly disagree” to 5=“strongly agree.”

*Health problem* was measured with the following question: “Have you ever experienced a health problem as a result of using the Internet information?” Response options ranged from 1=“never” to 5=“always.”

## Results

Our sample consisted of adults (n=710), almost equally distributed between men and women (381/710, 53.7% women), between the ages of 18 and 93 years with a mean of 48.82 ± 16.43. About 68% (481/710) were married, and 543 of 710 (77%) were Caucasian. Almost 40% (265/710) had a college degree or higher, and 405 of 710 (57%) earned $60,000 or more. Our comparison subsample consisted of respondents who were aged 60 years or older. They made up almost 30% (n=194) of our sample. About 40% (73/194, 37.6%) of them had a college degree or higher, and slightly more than half (99/194, 51.1%) reported an income level of $ 60,000 or more. Just over 60% were married (121/194, 62.4%), and a little over 80% (160/194, 82.5%) were Caucasian. We examined whether respondents’ sociodemographic characteristics and ehealth literacy were associated. There was a significant mean difference for the communication factor between men and women (2.10 vs 2.24, *P=*.047). There was also a racial or ethnic difference in means reported for the action factor with Caucasian participants reporting higher scores on the e-HLS (2.52 vs 2.35, *P=*.05). Significantly higher means were reported for the action and communication factors by those with higher education (2.20 vs 2.83, *P*=.001 for the action factor and 1.96 vs 2.34, *P=*=.001 for the communication factor). There is also a significant mean difference on the overall e-HLS score between respondents who had higher levels of education compared with those with lower levels (2.13 vs 2.77, *P*=.001). However, there was no statistically significant difference in the trust factor based on education (2.84 vs 2.71, *P=*.32). Respondents with higher income levels were also found to have a higher score on the overall e-HLS than those with lower incomes (2.52 vs 2.14, *P=*.01). We found a significant association between ehealth literacy and respondent age at neither the item nor factor level. Finally, married respondents had higher averages for the communication factor than nonmarried respondents (2.22 vs 2.08, *P=*.05). We examined bivariate associations of ehealth literacy with health-related variables in our survey. Respondents with higher scores on our measure of electronic health literacy reported higher sense of perceived empowerment (r=.395, *P*=.001), lower negative effect (worry or anxiety; r=-.116, *P=*.002), perceptions of more positive health care interactions with providers (r=.290, *P*=.001), and better health care communication (r=.427, *P*=.001). However, we also found a significant positive association with nonadherence (r=.454, *P*=.001) and experiencing a health problem as a result of using the Internet-based information (r=.128, *P*=.001). No significant associations were found between the ehealth literacy and perceived strain in health care interactions.

Next, we performed univariate examination of our scale items (Table 1). The item means for the full sample ranged from 1.93 ± 1.08 to 3.24 ± 0.95. The highest mean score was 3.24 ± 0.95 for the item that inquired about perceived confidence in ability to appraise information quality, followed by the item that asked about the extent to which the respondents believed that the Internet provides high-quality and credible health information (3.09 ± 0.75). The lowest average was found for the item that inquired whether respondents asked health professionals for advice about where to find credible information on the Internet (1.93 ± 1.08). Similar results were obtained for our subsample of older respondents. The highest mean scores were 3.23 ± 0.93 and 3.08 ± 0.74 for the aforementioned items. Similarly, the lowest average was for the item that questioned whether respondents asked health professionals for advice on where to find credible health information on the Internet (1.80 ± 0.98). The mean score of the composite scale was 2.51 ± 0.77 for the full sample, and it was 2.53 ± 0.81 for our subsample. Frequencies and percentages in Table 1 reflect the answers of those who responded “sometimes” ‘most of the time’ and “always.” Standard deviations are shown in parentheses.

Next, we performed the Kaiser–Meyer–Olkin (KMO) measure of sampling adequacy and Bartlett Test of Sphericity (BTS) to determine whether our data were suitable for EFA. Kaiser–Meyer–Olkin value of above 0.50 is needed before proceeding with EFA, whereas values of 0.80 or above are considered very good. A statistically significant BTS (*P=*.05 *)* indicates that sufficient correlations exist among the variables to proceed. The KMO and BTS results in our research indicated that the dataset satisfied the psychometric criteria for EFA analysis. Kaiser–Meyer–Olkin analysis yielded an index of 0.93, and BTS yielded 838.82, *P*=.001. We performed EFA with principal component analysis and varimax rotation using the following criteria: (1) eigenvalue greater than 1, (2) items loading on the same factor (≥0.30), (3) no crossloading, (4) Cattell’s scree test, and (5) conceptual interpretability of factors. Principal component analysis was chosen as a data extraction method because as Hair et al stated “This method focuses on extracting the minimum number of factors to account for the maximum portion of the total variance represented in the original set of variables’’ in the dataset [53]. The varimax rotation converged in 5 iterations. Three factors with eigenvalues greater than 1 emerged from the analyses. The eigenvalues for these factors were as follows: 8.52, 2.74, and 1.03. All the 3 factors explained 65% of the variance. A factor solution that accounts for 60% of the total variance is considered satisfactory [53].

Reestimation of the factor structure in our subsample confirmed this 3-factor solution. The varimax rotation converged in 4 iterations and provided the following eigenvalues: 9.17, 2.67, and 1.05. These factors explained 65% of variance in the data for the subsample of older adults. Although we used no crossloading as one of the criteria in determining the underlying factor structure of the e-HLS, we found that 2 items (perceived confidence to appraise information and discussing information with a health professional) crossloaded with 2 factors. We considered several alternative solutions to ensure that we had identified the best structure (1 less and 1 more factor than the initial solution suggested by EFA). We, then, determined to keep these 2 items in our composite scale because we deemed that they are conceptually important components of ehealth literacy. This decision is based on statisticians’ recommendation that “it is left up to the researcher to be the final arbitrator as to the form and appropriateness of a factor solution, and such decisions are best guided by conceptual rather than empirical bases” [53]. The distributions of the survey items to the factors in our full and subsample are summarized in Table 2 and Table 3, respectively.

**Table 1:** Univariate description of the e-HLS items.

Scale items		Means and standard deviations		Item frequencies and percentages	
		Full sample	Subsample	Full sample	Subsample
Action factor		2.48 (0.99)	2.52 (1.05)		
	Read disclosure statements	2.32 (1.20)	2.54 (1.20)	44.2% (311)	54.4% (105)
	Check credentials and affiliations of author	2.48 (1.36)	2.49 (1.42)	46.3% (327)	47.7% (92)
	Check who owns the website	2.41 (1.40)	2.22 (1.42)	43.8% (309)	34.2% (66)
	Check who sponsors the website	2.40 (1.37)	2.36 (1.42)	44.3% (311)	40.3% (76)
	Check if there is a financial tie between information and sponsor	2.20 (1.36)	2.25 (1.41)	36.4% (257)	36.3% (70)
	Appraise whether information provider’s credentials seem adequate	2.54 (1.40)	2.66 (1.42)	49.8% (348)	51.6% (99)
	Check whether an address is listed on the website	1.96 (1.09)	2.08 (1.21)	29.1% (205)	31.8% (61)
	Check whether goals and objectives of the website are clearly stated	2.26 (1.20)	2.34 (1.27)	41.4% (292)	42.9% (82)
	Appraise whether there is a clear and comprehensive coverage of the topic	2.63 (1.33)	2.75 (1.36)	54.2% (379)	55.6% (105)
	Check whether other print or Web resources confirm the information	2.57 (1.33)	2.57 (1.26)	51.5% (363)	51.3% (99)
	Check whether information is current and updated recently	2.90 (1.35)	2.91 (1.40)	61.5% (432)	60.6% (117)
	Check whether the last update of information is prominent on the website	2.66 (1.33)	2.68 (1.38)	53.4% (374)	51.8% (98)
	Confident of being able to appraise information quality on the Internet	3.24 (0.95)	3.23 (0.93)	80.8% (566)	77.6% (149)
Trust factor		2.79 (0.64)	2.78 (0.65)		
	Trust the Internet to provide accurate information	2.72 (0.86)	2.74 (0.87)	61.5% (432)	62.8% (120)
	Think information on the Internet as credible	3.09 (0.75)	3.08 (0.74)	84.5% (592)	85.4% (164)
	Think information on the Internet as balanced and accurate	2.95 (0.73)	2.94 (0.74)	79.2% (557)	78.1% (150)
	Think information on the Internet better than what most health providers supply	2.41 (0.87)	2.35 (0.87)	46.9% (328)	41.3% (79)
Communication factor		2.18 (0.90)	2.10 (0.87)		
	Discuss the information with a health provider	2.42 (1.07)	2.40 (1.06)	49.9% (311)	46.9% (90)
	Ask a health provider where to find credible information on the Internet	1.93 (1.08)	1.80 (0.98)	29.2% (206)	24.3% (47)

**Table 2:** Factor analysis of the full-sample e-HLS items.

Scale items	Full sample (n=710)			
	Factor I	Factor II	Factor III	Item total correlations
Read disclosure statements	0.66	0.01	0.26	0.60
Check credentials and affiliations of author	0.79	0.14	0.16	0.72
Check who owns the website	0.79	0.12	0.23	0.74
Check who sponsors the website	0.84	0.16	0.14	0.78
Check if there is a financial tie between information and sponsor	0.78	0.16	0.11	0.72
Appraise whether information provider’s credentials seem adequate	0.85	0.15	0.18	0.80
Check whether an address is listed on the website	0.76	0.05	0.23	0.70
Check whether goals and objectives of the website are clearly stated	0.79	0.03	0.13	0.74
Appraise whether there is a clear and comprehensive coverage of the topic	0.83	0.03	0.11	0.77
Check whether other print or web resources confirm the information	0.80	0.12	0.08	0.77
Check whether information is current and updated recently	0.85	0.08	0.05	0.77
Check whether the last update of information is prominent on the website	0.80	0.05	0.05	0.73
Confident of being able to appraise information quality on the Internet	0.45	0.32	0.43	0.43
Ask a health provider where to find credible information on the Internet	0.23	0.06	0.83	0.50
Discuss the information with a health provider	0.35	0.19	0.57	0.54
Trust the Internet to provide accurate information	0.34	0.75	0.01	0.34
Think information on the Internet as credible	0.17	0.86	0.01	0.20
Think information on the Internet as balanced and accurate	0.08	0.85	0.01	0.11
Think information on the Internet better than what most health providers supply	0.18	0.68	0.08	0.19

**Table 3:** Factor analysis of the subsample e-HLS items.

Scale items	Subsample: (n=194)			
	Factor I	Factor II	Factor III	Item-Total Correlations
Read disclosure statements	0.64	0.13	0.33	0.72
Check credentials and affiliations of author	0.81	0.02	0.12	0.73
Check who owns the website	0.85	0.08	0.03	0.79
Check who sponsors the website	0.86	0.01	0.06	0.81
Check if there is a financial tie between information and sponsor	0.82	0.04	0.06	0.76
Appraise whether information provider’s credentials seem adequate	0.89	0.01	0.07	0.84
Check whether an address is listed on the website	0.74	0.04	0.29	0.93
Check whether goals and objectives of the website are clearly stated	0.77	0.14	0.24	0.75
Appraise whether there is a clear and comprehensive coverage of the topic	0.83	0.11	0.10	0.78
Check whether other print or web resources confirm the information	0.81	0.02	0.16	0.80
Check whether information is current and updated recently	0.84	0.09	0.19	0.80
Check whether the last update of information is prominent on the website	0.78	0.07	0.22	0.75
Confident of being able to appraise information quality on the Internet	0.50	0.41	0.40	0.49
Ask a health provider where to find credible information on the Internet	0.36	0.03	0.78	0.49
Discuss the information with a health provider	0.47	0.17	0.55	0.58
Trust the Internet to provide accurate information	0.16	0.81	0.13	0.39
Think information on the Internet as credible	0.02	0.88	0.12	0.25
Think information on the Internet as balanced and accurate	0.09	0.86	0.08	0.09
Think information on the Internet better than what most health providers supply	0.06	0.72	0.15	0.18

On the basis of review of the existing literature, we labeled our first factor as *behavioral literacy (action factor).* It includes 13 items of behavioral indicators from the e-HLS. The factor loadings ranged from 0.45 to 0.85. The item that inquired if respondents appraised the adequacy of information providers’ credentials had the highest factor loading, whereas the item that asked if they were confident of their ability to appraise information quality on the Internet had the lowest factor loading. Similar patterns of factor item loadings emerged in our subsample with factor loadings ranging from 0.50 to 0.89. The factor mean is found to be 2.48 with a standard deviation of 0.99. We identified our second factor as *cognitive literacy (trust factor).* It consists of 4 items that assessed the perceived accuracy of health information on the Internet. The factor loadings ranged from 0.68 to 0.86. The item that assessed if they believed information provided on the Internet was credible had the strongest factor loading, whereas the item that assessed if they thought information provided on the Internet was the same as or better than what most health professionals provided had the lowest factor loading. Similar factor loading patterns were found for our comparison subsample, with factor loadings ranging from 0.72 to 0.88. The factor mean is 2.79, and standard deviation is 0.64. We identified our third factor as *interactional literacy (communication factor)*. It consists of 2 items that measure the extent to which discussion of Internet information takes place between health care provider and information user. The factor loadings ranged from 0.57 to 0.83. The item that assessed if respondents asked health professionals for advice about where to find credible information on the Internet showed the strongest factor loading, whereas the item that measured if they discussed information obtained from the Internet with a health professional showed the lowest factor loading. A similar pattern emerged with our comparison subsample with factor loadings ranging from 0.55 to 0.78. The mean value for this factor is 2.18 with a standard deviation of 0.91.

Of the 3 factors, the trust factor has the highest mean score (2.79 ± 0.64 for the full sample; 2.78 ± 0.65 for the subsample). The action factor has the next highest mean scores (2.48 ± 0.99; 2.52 ± 1.05). The communication factor has the lowest averages for the full sample and the subsample (2.18 ± 0.90; 2.10 ± 0.87). When the correlations between the factors were examined, low-to-moderate to moderate-to-high significant associations emerged. In the full sample, the action and communication factors have the highest correlation with each other (r=.59, *P*=.001) with the trust factor correlating with both communication and action factors (r=.21, *P*=.001 and r=.17, *P*=.001). Similar results were obtained for our subsample. The action and communication factors have a high correlation with each other (r=.60 at *P*=.001) and the trust factor correlating with both communication and action factors (r=.23, *P*=.001 and r=.17, *P*=.001).

**Figure 1:**
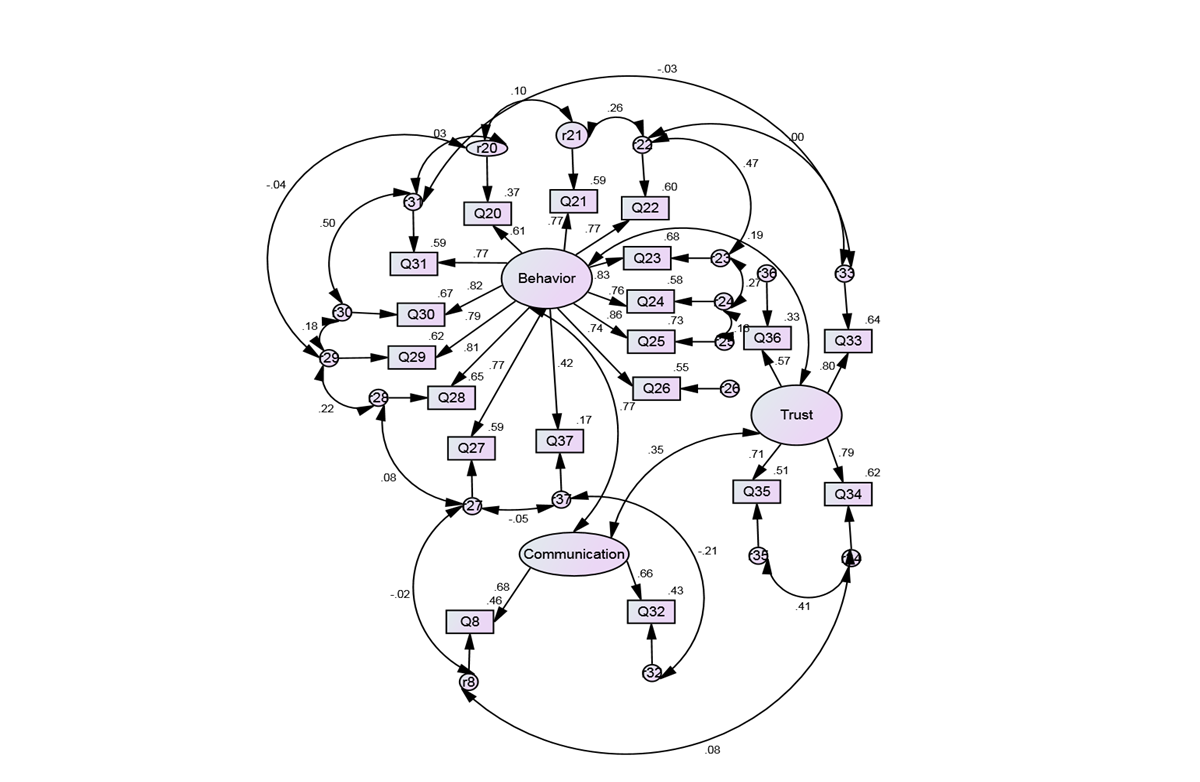
Confirmatory Factor Analysis of the e-HLS Items (n=710).

**Figure 2:**
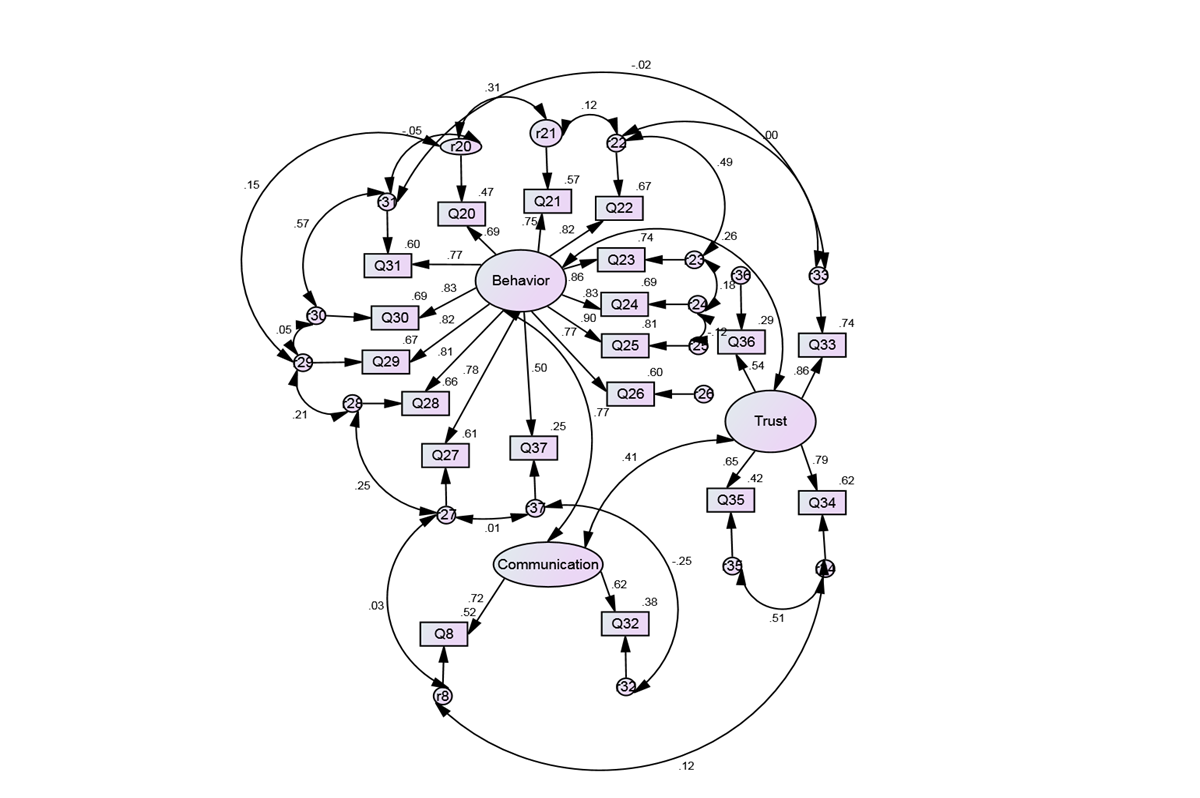
Confirmatory Factor Analysis of the e-HLS Items (n=194).

Confirmatory factor analysis using AMOS 21 statistical program verified that the 3-factor structure of the e-HLS is invariant for the full and subsample and achieved a good fit with both. Comparative fit index and NFI values close to 1 and RMSEA index less than 0.10 are generally deemed to be a good fit. Fit indices obtained for the full sample were as follows: chi-square (710)=698.547, *df*=131, *P*=.001, CFI=0.94, NFI=0.92, RMSEA=0.07. All factor loadings were significant (*P*<.001) with standardized regression coefficients exceeding 0.40. The estimates for the older subsample were as follows: chi-square (194)=275.744, *df*=131, *P*=.001, CFI=0.95, NFI=0.90, RMSEA=0.08. All factor loadings were also significant (*P*<.001) with standardized regression coefficients exceeding 0.50. The significant goodness-of-fit value given by the chi-square index was likely a result of the sample size because as sample size increases, the chi-square value quickly approaches significance and should not be interpreted as an indication of poor model fit [72,73]. The output path diagram showing the computed values for the entire sample is depicted in Figure 1, and that of the subsample is depicted in Figure 2. Finally, internal consistency reliability analysis of the e-HLS demonstrated high Cronbach alpha values: .93 for the full sample and .94 for our subsample.

Next, we examined the validity of our measure by performing external correlates test. According to DeCoster, ‘‘you can (and should) assess validity in a number of different ways. Each time you demonstrate that the scale acts in a way consistent with the underlying construct you make a more convincing argument that the scale provides an accurate representation of that construct’’ [71]. The typical scale validation involves assessing the newly developed scale as it relates to other constructs. Spector stated, “[t]he typical scale-validation strategy involves testing the scale of interest in the context of a set of hypothesized interrelations of the intended construct with other constructs’’ [56]. To confirm the validity of our new scale, we needed to assess how it associated with related constructs. For this purpose, therefore, we performed both bivariate and multivariate analyses using our full sample and subsample.

First, we examined bivariate associations of ehealth literacy with health-related variables in our survey. Respondents with higher scores on our measure of electronic health literacy reported a higher sense of perceived empowerment (r=.395, *P*=.001), less negative effect (worry or anxiety; r=-.116, *P=*.002), perceptions of more positive health care interactions with providers (r=.290, *P*.001), and better health care communication (r=.427, *P*=.001). However, we also found a significant positive association with nonadherence (r=.454, *P*=.001) and experience of a health problem as a result of using Internet-based information (r=.128, *P*=.001). No significant association existed with perceived strain in health care interactions. Our eHealth literacy instrument also revealed that higher scores on our scale were correlated with education (r=.20, *P*=.001) and income (r=.10, *P*=.01). In regard to the subsample, we found that our instrument displayed the following correlations: older respondents who obtained higher scores on our instrument reported a higher sense of perceived empowerment (r=.502, *P*=.001), perceptions of more positive health care interactions with providers (r=.304, *P*=.001), and better health care communication (r=.489, *P*=.001). We also found a significant positive association with nonadherence (r=.533, *P*=.001), strained interactions with health providers (r=-.176, *P*=.01), and less negative effect (r=-.152, *P*=.001). In our subsample, higher scores on our ehealth literacy scale were negatively correlated with older age (r=-.167, *P*=.02) and positively correlated with education (r=.296, *P*=.001).

We also performed linear regression analyses to examine the extent to which the factorial structure of the e-HLS was differentially associated with health variables from our survey dataset. Consistent with the literature cited in the beginning of this paper, we chose the following variables to run our multivariate regression analyses: health interaction, health communication, nonadherence, perceived empowerment, negative effect, and health problem. As summarized in Table 4 and Table 5 (numbers are rounded up), the regression coefficients for the action factor for the full sample were significant for the following variables after controlling for the effect of demographic variables: perceived empowerment (β=.303, *P*=.001), nonadherence (β=.316, *P*=.001), health communication (β=.206, *P*=.001), and negative effect (β=-.174, *P*=.001). For our subsample, we found the action factor to be significantly associated with health communication (β=.140, *P*=.053), perceived empowerment (β=.365, *P*=.001), negative effect (β=-.250, *P*=.005), and nonadherence (β=.312 *P*=.001). The action factor had no significant association with positive health interaction, strained health interaction, and health problem. The *b* represents unstandardized regression coefficients (slope), and *β* represents standardized regression coefficients. The regression coefficients for the trust factor for the full sample were significant for the following variables: perceived empowerment (β=.293, *P*=.001), nonadherence (β=.216 *P*=.001), positive health interaction (β=.282, *P*=.001), health communication (β=.280, *P*=.001), strained health interaction (β=.092, *P*=.02), and negative effect (β=.077, *P*=.04). Significant associations for the trust factor in the subsample of older adults include positive health interaction (β=.340, *P*=.001), health communication (β=.326, *P*=.001), perceived empowerment (β=.299, *P*=.001), and nonadherence (β=.249, *P*=.001). The trust factor has no significant association with reports of a health problem at the multivariate level. The significant coefficients for the communication factor after controlling for the effects of demographic coefficients include the following variables: perceived empowerment (β=.106, *P*=.001), nonadherence (β=.191, *P*=.001), positive health interaction (β=.308, *P*=.001), health communication (β=.323, *P*=.001), and health problem (β=.147, *P*=.002). In the subsample, significant associations emerged with positive health interaction (β=.238, *P*=.001), health communication (β=.350, *P*=.001), and nonadherence (β=.206, *P*=.001). There were no significant associations with strained health interaction in the full sample or subsample.

**Table 4:** Ordinary least regression analysis of the e-HLS factorial structure for the full sample (n=710).

	Health interaction		Health communication		Strain		Empowerment		Negative affect		Health problem		Nonadherence	
e-HLS Factors	*b*	β (*P*)	*b*	β (*P*)	*b*	β (*P*)	*b*	β (*P*)	*b*	β (*P*)	*b*	β (*P*)	*b*	β (*P*)
Action	0.055	.077 (.06)	0.163	.206 (.001)	-0.027	-.033 (.46)	0.254	.303 (.001)	-0.153	-.174 (.001)	0.013	.032 (.49)	0.193	.316 (.001)
Communication	.235	.302 (.001)	0.279	.323 (.001)	0.017	.019 (.68)	0.097	.106 (.001)	0.072	.075 (.11)	0.067	.147 (.002)	0.128	.191 (.001)
Trust	.308	.282 (.001)	0.340	.280 (.001)	0.116	.092 (.02)	0.377	.293 (.001)	0.077	.077 (.04)	-0.007	-.011 (.78)	0.204	.216 (.001)
R^2^		.247		.359		.009		.266		.025		.012		.292
Adjusted R^2^		.243		.356		.005		.263		.021		.023		.289

**Table 5:** Ordinary least regression analysis of the e-HLS factorial structure for the subsample (n=194).

	Health interaction		Health communication		Strain		Empowerment		Negative affect		Health problem		Nonadherence	
e-HLS Factors	*b*	β (*P*)	*b*	β (*P*)	*b*	β (*P*)	*b*	β (*P*)	*b*	β (*P*)	*b*	β (*P*)	*b*	β (*P*)
Action	0.016	.024 (.77)	0.098	.140 (.05)	-0.121	-.165 (.07)	0.273	.365 (.001)	-0.191	-.250 (.005)	0.027	.109 (.24)	0.187	.312 (.001)
Communication	0.193	.238 (.004)	0.296	.350 (.001)	0.015	.017 (.85)	0.053	.059 (.45)	0.072	.078 (.38)	0.002	.006 (.95)	0.149	.206 (.007)
Trust	0.364	.340 (.001)	0.364	.326 (.001)	-0.089	-.075 (.31)	0.356	.299 (.001)	0.137	.112 (.13)	-0.034	-.088 (.24)	0.239	.249 (.001)
R^2^		.220		.375		.033		.298		.052		.017		.329
Adjusted R^2^		.207		.365		.018		.286		.037		.001		.319

## Discussion

The Institute of Medicine’s recommendation to expand the scope of health literacy by considering multiple skills has led to increased recognition that a comprehensive examination of health literacy in the digital environment is needed [18,66-75]. Our research study is also consistent with the National Call to Action to Promote Health Literacy, which suggested the need to develop multidimensional measures of health literacy to include skills beyond the comprehension of written health information [76].

The strength of our measure is its contemporary multidimensional view of health literacy. We expand its conceptualization beyond the traditional document-based measures (being able to find and understand information) to include interactive and communicative aspects of literacy (information exchange) and critical evaluative skills of information (quality assessment) provided in electronic sources. We created our instrument to comprise of 3 domains: behavioral literacy (action factor), cognitive literacy (trust factor), and interactional literacy (communication factor). Thus, our measure expands the understanding of ehealth literacy through the addition of the 3 domains. Because the concept of health literacy is increasingly conceptualized as consisting of skills related to evaluating, communicating, and using information to make informed decisions, we designed our new measure to reflect these skills.

The results provide statistical support for the multidimensionality of our scale. Consistent with recent studies, our scale suggest that ehealth literacy includes a broader array of skills besides the ability to read and understand health information stressing the need to focus on multiple dimensions of content areas and skills [30,33,77]. The ability of this composite scale to provide information about the extent to which people assess the quality and credibility of ehealth information makes it a valuable assessment tool. Although the composite scale yields a single score, combining data from items that are loaded onto separate factors into a single score may suppress potential differences that can be found if the scale factors are analyzed separately. In our study, we first evaluated the psychometric properties of a new ehealth literacy measure in a national sample of Internet users. Confirmatory factor analysis was conducted to determine whether the 3-factor structure of the e-HLS, as suggested by EFA, achieved a good fit with our entire sample and subsample. The goodness-of-fit indices provided by CFA confirmed the robustness of the e-HLS. Each of the scale factors demonstrated a good internal consistency and validity. We examined patterns of correlations of our measure with related covariates. Moreover, we regressed scale factors on variables that literature has shown to be associated with health literacy in the general sample [51].These analytical approaches further validated our new measure. However, some limitations in our study must be acknowledged. First, we had to rely on self-reported cross-sectional data, and we lacked useful information illustrating the extent to which our respondents used the Internet for health information. Furthermore, we were not able to measure health literacy to its fullest dimensions, as a wide range of skills and behaviors comprise health literacy. Moreover, as a means of evaluating health literacy, self-report may not be entirely accurate. Regardless, this method may further improve our understanding of the role of health literacy in the daily lives of Americans and provide constructive information to that end.

As summarized in Table 1, the fact that our respondents reported high confidence in discerning information quality and trust in Internet information while reporting low rates of behavior to verify information credibility and quality suggests low awareness about the questionable trustworthiness and credibility of information found on the Internet. Communication with health professionals for purposes of asking advice about which websites they should consult and where to find credible information on the Internet was not common either. As patients want a greater understanding and more active role in in their health management, we need new and expanded approaches to examine health literacy to incorporate the provision of credible information through the new digital technologies [77,78]. When examining indicators of electronic health literacy among our respondents, we found higher scores among those with higher levels of education and income. The factorial structure of our scale explained the greatest variance for respondents’ perceived positive changes in health communication with their providers followed by patient nonadherence, perceived empowerment, and positive health interaction. On the other hand, the factor structure of our scale had little explanatory power for perceived strain in health interactions, negative effect, and experience of a health problem. Our results suggest that satisfaction with medical encounters is enhanced by consumers with higher levels of ehealth literacy.

Examination of standardized beta coefficients revealed that the trust factor of our measure had a strong association with perceived empowerment, suggesting that those respondents who trusted information gathered from the Internet to a greater extent also reported higher sense of empowerment. Consistent with existing research, around 60% of our respondents reported relatively little skepticism of the quality of Internet health information despite the fact that much of the Internet health or medical information is of questionable accuracy and lacks any endorsement or sanction of by a formal medical authority [63,70,79]. This is particularly surprising given that less than half of our participants reported having performed a quality check of the Internet information on most of our scale items. We found that they trusted information provided by health websites, and they overestimated the credibility and accuracy of information presented. This is despite various challenges associated with the Internet search, including information overload, navigating through hundreds of search results, many of which could be irrelevant, and separating questionable from credible health information. Moreover, face-to-face, traditional medical encounters may become awkward or strained now that health care professionals no longer enjoy an information monopoly. The way patients can engage with health information, including accessing information, about unverified alternative medicines, may pose challenges for doctors and other providers who need to interact personally with consumers who can now gather their information from the Internet. This actually may form the crux of the problems troubling health professionals; patients exercise their elevated sense of health care empowerment yet remain insufficiently cognizant of the real dangers of trying to manage their health based on inconsistent and potentially highly inaccurate Web-based information. Health care professionals’ fears may be well founded. Our regression analyses indicated that information seekers who place a great deal of trust in Internet information report greater levels of nonadherence. In addition, the trust in Internet information (trust factor) is significantly associated with patient nonadherence to doctor’s guidelines and/or treatments, further confirming professionals’ concerns.

Not surprisingly, a high level of trust in Web-based information had a significant positive association with strained interactions during medical encounters as reported by our research participants. Interestingly, although we found a significant association between trust in the Internet information and reports of perceived strain in medical encounters in our full sample, this association was not significant in the subsample. This might suggest that health professionals were less likely to feel challenged or distrusted by older patients using the Internet to seek health or medical information about their concerns than younger patients. In addition, the action factor had a negative significant association with negative effect (worry and/or anxiety), whereas the trust in Internet factor had a positive association with negative effect. These findings suggest that respondents who took action to evaluate health information reported less worry and/or anxiety, whereas those who placed a great deal of trust in the Internet information reported more worry and/or anxiety.

The action factor of our scale explained the most variance in perceived empowerment and nonadherence. Respondents who engaged in various quality checks of health information seem to perceive themselves as better equipped to cope with their health concern or issue. On the other hand, the communication factor had the highest explanatory power for positive health interaction and health communication. These associations suggest that health consumers who work with their health care professionals to find the most credible sources before they search the Internet perceive positive changes in their encounters with their providers. When patients share the information they discovered on the Web with their providers, they ask more informed questions and better understand the doctor’s information. Moreover, they perceive respect from their providers as partners in the health care process.

In contrast, perceived sense of empowerment, as a result of information obtained from the Internet sources, without communication with a health care provider is associated with increased rates of noncompliance with treatment and medical advice of a health professional. Accordingly, further examination of sociotechnological changes and their effect on doctor–patient interaction and communication is warranted.

## Abbreviations

BTS: Bartlett Test of Sphericity
CFA: confirmatory factor analysis
CFI: comparative fit index
EFA: exploratory factor analysis
e-HLS: electronic health literacy
KMO: Kaiser–Meyer–Olkin
KN: Knowledge Networks
NFI: normed fit index
RMSEA: root mean squared error of approximation
